# Epistemically exploitative bullshit: A Sartrean account

**DOI:** 10.1111/ejop.12810

**Published:** 2022-07-03

**Authors:** Thomas Szanto

**Affiliations:** ^1^ Center for Subjectivity Research, Department of Communication University of Copenhagen Copenhagen Denmark

## Abstract

This paper presents a novel conceptualization of a type of untruthful speech that is of eminent political relevance but has hitherto been unrecognized: epistemically exploitative bullshit (EEB). Speakers engaging in EEB are bullshitting: they deceive their addressee regarding their unconcern for the very difference between truth and falsity. At the same time, they exploit their discursive victims: they oblige their counterparts to perform unacknowledged and emotionally draining epistemic work to educate the speakers about the addressees' oppression, only to discredit their epistemic trustworthiness. I argue that EEB is irreducible to various recently discussed untruthful speech, and in particular to Frankfurtian bullshit, as well as to epistemic exploitation or other epistemic injustices. Taking inspiration from Sartre's analysis of anti‐Semitic discourse, where bullshitting and epistemic exploitation are essentially interlinked, I instead suggest that recognizing the distinctiveness of EEB allows for a more refined conceptualization of these discursive phenomena. Specifically, I show how bad faith and the ensuing collective diffusion and delegation of epistemic responsibility play a so far neglected but key role here. I ultimately demonstrate that with Sartre's help, we can grasp how the existential, interpersonal and institutional dimensions involved in the negotiation of truth seamlessly intersect better than we would with the lens of analytic or critical epistemology alone.

“Never believe that anti‐Semites are completely unaware of the absurdity of their replies. They know that their remarks are frivolous, and open to challenge. But they are amusing themselves, for it is their adversary who is obliged to use words responsibly since he believes in words. The anti‐Semites have the right to play. They even like to play with discourse for, by giving ridiculous reasons, they discredit the seriousness of their interlocutors. They delight in acting in bad faith since they seek not to persuade by sound argument but to intimidate and disconcert. (…) If then (…), the anti‐Semite is impervious to reason and to experience, it is not because his conviction is strong. Rather his conviction is strong because he has chosen first of all to be impervious.”

 (Sartre 1995b[1946], 19–20)

## 1 INTRODUCTION

The acquisition, sincere expression and communicative dissemination of truth are epistemic virtues. Bernard Williams (2002) famously subsumed these virtues under the concept of “truthfulness.” Williams' originality lies in showing how the question of the nature of truth is essentially linked to its social, moral, and political value. For Williams, truth has a value that cannot be grasped truth‐functionally or assessed solely by determining the truth‐value of assertions. The value of truth has rather do with respect for and trust in truthful speech.

Truthfulness, for Williams, is in turn characterized by two particular epistemic virtues, which we might call “alethic cardinal virtues”: accuracy and sincerity. Accuracy refers to the diligence or care in the acquisition of true beliefs; sincerity is the disposition to say only what one believes to be true, or at least not to say what one believes to be false.^1^ The two cardinal virtues can be summed up by the following imperative: “Do your best to acquire true beliefs, and when you communicate your beliefs to others, reveal what you believe!”; call this “William's imperative” (cf. Williams, 2002, p. 11). Whereas accuracy involves epistemic reliability, the disposition of being sincere is tied to the epistemic trustworthiness of speakers: Hearers can, by and large, trust that what sincere speakers say is what they believe to be true and that these beliefs will, by virtue of the accuracy of speakers' information‐gathering, typically be, in fact, true (Williams, 2002, 93ff.).

But truthfulness is just as much an epistemic as a political virtue. Arguably, this is more obvious today than ever before. Yet truthfulness as a political virtue involves far more than the platitudes about the impact of fake news on politics in the so‐called post‐truth era,^2^ but also more than what Williams's imperative alone indicates.

In this paper, I draw attention to an irreducible and eminently political form of speech that has hitherto remained virtually unrecognized, but which lies at the basis of and biases quite ordinary communicative exchanges. I argue that the form of speech in question is not so much a direct violation as a strategic subversion of Williams' imperative: The speaker does nothing to acquire and communicate true beliefs. On the contrary, he does everything to deceive his interlocutors regarding his utter indifference as to whether his beliefs are true or false, or what I call his “generalized alethic insouciance.” Moreover, the speaker epistemically exploits his interlocutors. He tries to obtain certain information from them, only to dismiss these and discredit the epistemic trustworthiness of his interlocutors, thus cementing existing asymmetric discursive positions and his own communicative dominance.

This is what I call *epistemically exploitative bullshit* (EEB). I introduce this admittedly cumbersome term to refer to two central epistemological debates that have so far run isolated from each other, to the detriment of both: On the one hand, the discussion of what since Frankfurt (1986) has become known as “bullshit” and related work from analytic epistemology on cognate types of mendacious speech; on the other hand, the recently flourishing literature from critical epistemology on epistemic injustice and oppression, in the context of which my focus, epistemic exploitation (Berenstain, 2016), has been discussed.

But long before these debates, Sartre conceived of anti‐Semitic discourse as a form of EEB in his 1945 book *Anti‐Semitism and Jew*, or so I suggest. As far as I'm aware, Sartre is indeed the only author to have duly considered this phenomenon. But regardless of the uniqueness of his proposal, my aim here is not to reevaluate Sartre from a critical epistemology perspective.^3^ Rather, taking inspiration from Sartre, I want to show, first, that EEB is irreducible to other epistemically vitious discourse, in particular, to bullshit or epistemic exploitation, and, second, that recognizing the distinctiveness of EEB allows for a better understanding of both bullshit and epistemic exploitation. More generally, I suggest that conceptualizing EEB is indispensable for understanding certain pervasive forms of politically motivated subversion of communication, the cementing of discursive positions of power and concomitant discursive antagonisms.

I start by elaborating on Frankfurt's notion of bullshit as a form of non‐alethic, or non‐truthful, speech that needs to be distinguished from conventional forms of lying.^4^ In the next section, I turn to the phenomenon of epistemic exploitation and characterize it as a form of epistemic injustice and oppression. In the last main section, I present my Sartrean conception of EEB and I show that, while it integrates mechanisms of both bullshit and epistemic exploitation, it is a *sui generis* type of non‐truthful speech. Moreover, I show that Sartre is more nuanced in his account of the deception involved than current accounts are. The bullshitter's supposedly casual insouciance for the very difference between truth and falsity, for Sartre, is radicalized in the self‐deceptive insincerity of bad faith and a certain “alethic *ur*‐anxiety,” which lies at the bottom of epistemic exploitation. I argue that the result is a flight from the responsibility for the value of truth, and eventually the collective diffusion of epistemic responsibility and its forced delegation to the oppressed. I will ultimately demonstrate that with Sartre, we can grasp how the existential, interpersonal and institutional dimensions in the negotiation of truth, knowledge, and power seamlessly intersect better than with the lens of analytic or critical epistemology alone.

## 2 ALL LIES AND DECEPTION? BULLSHIT AND CONVENTIONAL LIES

The paradigm against which all non‐alethic speech must be gauged is, quite obviously, conventional lying.^5^ According to the standard view, a conventional lie is a statement that the speaker believes to be false and asserts with the intention of deceiving the addressee into believing it to be true. More precisely, according to a still widely held definition, a lie must meet the following conditions (see Mahon, 2015):

1. *Assertion Condition*: A speaker S makes a statement *p*.
2. *Untruth Condition*: S believes *p* to be false.
3. *Addressee Condition*: There is an addressee A to whom *p* is addressed.
4. *Intentional Deception Condition*: S has the intention that A believes *p* to be true.

The first thing to note is that this definition does not require that the intention to deceive is in fact fulfilled or that the deception is successful. Thus, A need not actually believe *p* to be true for S to count as a liar. Some, however, think that (2) must be qualified such that the statement *p* must in fact be false (Carson, 2006; Turri, 2021). But most consider such a qualification of the untruth condition too narrow, and it indeed places rather high epistemic demands upon a liar. Accordingly, most hold that a liar only needs to believe *p* to be false (Fallis, 2009). Whichever view one favors, bullshit must clearly be distinguished from either conception of conventional lies.

Specifically, according to Frankfurt's original account,^6^ bullshit must be distinguished from conventional lies in two respects: first, by the relationship that the liar or the bullshitter adopts to the truth/falsity of their statements, and second, with reference to the different form of deception involved. Thus, bullshit can be determined by modifying the untruth (2) and the intentional deception condition (4).

As we have seen, standardly conceived, the liar believes a relevant statement *p* to be false (2), which means that he believes that he knows the truth value of his statement. As Frankfurt puts it, “it is impossible for someone to lie unless he thinks he knows the truth.” (1986, p. 55) To intentionally make a *false* assertion, then, the liar must have some concern for the truth value of his statements. This concern for truth will typically be minimal. The liar will not bother to obtain and verify all available information that would justify his statement (were he to tell the truth). He certainly will not worry about the truth of his statement in the sense of Williams' accuracy condition. Nevertheless, he cannot be completely unconcerned whether his statement, which he deceptively represents as true, is, in fact, true or false.

Herein lies the first crucial difference between the bullshitter. In contrast to the liar's concern for truth, bullshit is characterized by a fundamental unconcern for the very difference between truth and falsity, or what I call “generalized alethic insouciance.”^7^ It's not just that the bullshitter does not give a damn about whether his particular statements are true or false; the very difference between true and false statements, in general, becomes irrelevant in bullshitting. Thus, while the liar “rejects the authority of truth” and “refuses to meet its demands,” but is still “guided by” these, “the bullshitter ignores these demands altogether.” The epistemic unconcern of the bullshitter is by no means harmless, and Frankfurt rightly emphasizes that bullshit is “a greater enemy of the truth than lies are” (1986, pp. 60–61).^8^

The most precise characterization of alethic insouciance in bullshit has been proposed by Stokke and Fallis. According to them, “A is bullshitting relative to a [question under discussion] *q* if and only if A contributes *p* as an answer to *q* and A is not concerned that *p* be an answer to *q* that her evidence suggests is true or that *p* be an answer to *q* that her evidence suggests is false.” (Stokke & Fallis, 2017, p. 295) The advantage of this characterization is that it captures the speaker's alethic insouciance as an indifference to whether his contribution to a question under consideration is grounded in any attempt at justifying what he says or reflects his evidence, rather than as an indifference regarding the truth‐value of his assertions. As such, the characterization is well‐suited to capture cases of bullshit brought up against Frankfurt, in which the bullshitter is precisely not indifferent to the truth of what he says (Carson, 2010; Kimbrough, 2006; Wreen, 2013; cf. Fallis,  2015b). Indeed, a bullshitter may well be interested in the truth‐value of their statements and wish or hope that those turn out to be true. Consider an ardent conservative, who, based on a Fox News report^9^ full of obviously distorting statistics, repeatedly proclaims in discussions about the Scandinavian welfare‐state model that Denmark is in just as miserable a socio‐economic situation as Venezuela, and that one can see from this where “state socialism” leads. Assuming that our Fox News fan is interested in the forcefulness of his arguments against alleged machinations of state socialism, he will certainly not be unconcerned whether his statements regarding the socio‐economic position of Denmark vis‐à‐vis Venezuela are correct.^10^ Moreover, as Stokke and Fallis also point out, he will care about disseminating statements that promote his beliefs, beliefs whose truth are indeed a concern of his. But assuming he sees through the caricatured nature of the evidence presented, he is still bullshitting, insofar as he is unconcerned whether his statements about state socialism are supported by any evidence.

Now, like many others, Stokke and Fallis disregard the second^11^ and, according to Frankfurt (1986, p. 54), “only indispensably distinctive characteristic” of bullshit, namely a modified version of the intentional deception condition (a condition that, yet modified, will also be central for EEB). The liar, we recall, knowingly and deliberately deceives the addressee by presenting a statement *p*, that he believes to be false, as true. But the liar primarily deceives the addressee about the truth value of the statement and only indirectly as to what he is doing, namely lying. If the intention to deceive is successful and the addressee takes the statement to be true, the addressee will not perceive the lie as a lie and be automatically misled regarding the liar's activity. And yet, the liar aims not so much to deceive the addressee about the mendacious nature of the exchange of information but, rather, about the accuracy of the information itself, or the truth of a statement. As Frankfurt also points out, “the success” of a lie (as well as of bullshit) “depends upon [the liar hiding that] he is attempting to lead us away from a correct apprehension of reality; we are not to know that he wants us to believe something he takes to be false.” And in doing so, the liar “represents [himself] falsely as endeavoring to communicate the truth.” (1986, pp. 54–55). But again, the primary goal of the liar's deceptive intent is not to conceal that he is lying but, rather, how things stand with what this activity purports to represent. Concealing the untruthful nature of the activity is more like an after‐effect of lying.

The exact opposite happens in bullshit. An essential characteristic of bullshit is that the speaker aims to “deceive us about [his] enterprise” (Frankfurt 1986, p. 54). The bullshitter “misrepresents what he is up to” (Frankfurt 1986, p. 54), but does not, like the liar, necessarily misrepresent how it stands with the things about which he produces bullshit. To grasp the difference between the “discursive falsity” of the bullshitter, as we may call it, and the truth‐functional falsity of the liar, Frankfurt compares the deception of the bullshitter with the deception in a forgery or faking, such as a fake art piece:

“Unlike plain lying, [bullshit is] a matter not of falsity but of fakery. […]. For the essence of bullshit is not that it is *false* but that it is *phony*. […] What is wrong with a counterfeit is not what it is like, but how it was made. This points to a similar and fundamental aspect of the essential nature of bullshit: although it is produced without concern with the truth, it need not be false. The bullshitter is faking things. But this does not mean that he necessarily gets them wrong.” (Frankfurt, 1986, pp. 47–48; see also Frankfurt, 2006, pp. 3–4)

But what exactly is fake in bullshit, or what is it that the bullshitter tries to deceive others about? It is what I have called generalized alethic insouciance. As Frankfurt explains: “The fact about himself that the bullshitter hides […] is that the truth‐values of his statements are of no central interest to him; what we are not to understand is that his intention is neither to report the truth nor to conceal it.” (1986, p. 56) Frankfurt emphasizes that “this does not mean that his speech is anarchically impulsive, but that the motive guiding and controlling it is unconcerned with how the things about which he speaks truly are.” (1986, p. 56) That bullshit is not simply anarchic is a crucial point. The bullshitter is not simply epistemically wavering or discursively “impulsive,” as if he merely presented one and the same fact ad lib once as true, once as false. Rather, his speech is motivated. Indeed, the cases of bullshit that interest us have a strategic function. As I will show, we can understand the strategic character of those types of bullshit that represent a discursive subversion only in connection with the epistemic exploitation peculiar to them. For the time being, let us note that certain cases of bullshit are not merely epistemically insouciant but are, rather, *subverting* the *ethics* of discourse.

In summary, there are two features of Frankfurtian bullshit that clearly distinguish it from all other forms of non‐alethic speech^12^: first, bullshitters often assert something false, but, in contrast to lies, neither does this wittingly nor necessarily so. Secondly, there is a specific intention to deceive: the bullshitter aims to deceive the addressee as to her own generalized alethic insouciance.

However, as should become clear in the next section, the discussion on bullshit suffers not only from being one‐sidedly oriented towards the question of how it relates to lying and the falsity and deception ordinarily involved therein^13^; more importantly, the discussion remains too narrowly focused on *dyadic* or *interpersonal* communication, assuming a single speaker and one or more individual addressees. What is sorely missing is any reference to the broader communicative community and, in particular, an analysis of the discursive power structures into which these forms of speech are often embedded. Such an analysis, however, is indispensable for understanding those important forms of bullshit that not only subvert rational discourse and inhibit the ability to effectively share information but also aim at eroding trust in the credibility of the communication partners.^14^ What is underappreciated is, therefore, the broader, discursively strategic aim of some forms of bullshit to establish or maintain power‐asymmetries, an aim they share with epistemic exploitation.^15^

## 3 EPISTEMIC EXPLOITATION

In her blog entry, “Why I'm No Longer Talking to White People About Race”^16^ from February 22, 2014, the British journalist Reni Eddo‐Lodge writes:

“[…] I can't talk to white people about race anymore because of the consequent denials, awkward cartwheels and mental acrobatics that they display when this is brought to their attention. Who really wants to be alerted to a structural system that benefits them at the expense of others? […] I can't have a conversation with them about the details of a problem if they don't even recognize that the problem exists. […] Not to mention that entering into conversation with defiant white people is a frankly dangerous task for me. As the heckles rise and the defiance grows, I have to tread incredibly carefully, because if I express frustration, anger or exasperation at their refusal to understand, they will tap into their pre‐subscribed racist tropes about angry black people who are a threat to them and their safety. It's very likely that they'll then paint me as a bully or an abuser. […] It's truly a lifetime of self‐censorship that people of color have to live. […] I cannot continue to emotionally exhaust myself trying to get this message across […] I don't have a huge amount of power to change the way the world works, but I can set boundaries. I can halt the entitlement they feel towards me and I'll start that by stopping the conversation. The balance is too far swung in their favor. Their intent is often not to listen or learn, but to exert their power, to prove me wrong, to emotionally drain me, and to rebalance the status quo.” (Eddo‐Lodge, 2017, pp. x–xii)

This excerpt aptly captures all essential features of the discourse situation at stake, which Berenstain (2016) refers to as “epistemic exploitation.”^17^

Epistemic exploitation is a form of the epistemic‐discursive asymmetry discussed in terms of epistemic injustice in the wake of Fricker's seminal work (Fricker, 2007).^18^ Most broadly, epistemic injustice refers to various structural practices by individuals and institutions, such as the judiciary or the workplace, that are both dysfunctional with respect to epistemic values such as truth, correctness, or understanding, and unfair or oppressive towards those individuals or groups who possess and communicate a particular body of knowledge (cf. Pohlhaus, 2017, p. 13). Such practices include not only the outright exclusion or silencing of certain speakers but also more subtle ways of diminishing their epistemic and communicative status and marginalizing their contribution. One important example of this, so‐called testimonial injustice, is to truncate or disregard the testimony of minority speakers or credit them less than an average majority speaker.^19^ What we already recognize here is those epistemic injustices are not only epistemically, let alone merely truth‐functionally, disruptive like the above‐discussed non‐alethic forms of speech. They are also socially unjust, namely to *specific communities* of addressees and not to some abstract or general placeholders of these (“an addressee X”).

Epistemic injustice is never an isolated or momentary occurrence of injustice. It inevitably involves a deliberate or accepted perpetuation of injustices. It thus engenders structural or strategically employed processes of oppressing the unjustly treated. Dotson, who has most systematically conceptualized epistemic oppression, defines it as a “persistent” and “unwarranted infringement” on the ability of discourse participants to “utilize persuasively shared epistemic resources,” thus impeding the “revision” of these resources if required and “hinder[ing] one's contribution to knowledge production” (Dotson, 2014, p. 115). The oppressed, then, are doubly excluded as epistemic agents: not only is their participation in shared epistemic resources prevented, their potential contribution and correction to a shared pool of knowledge is also excluded beforehand.^20^

But to what extent is epistemic oppression *epistemically* unjust and oppressive, and thus irreducible to generally prevailing forms of social or political injustice or oppression (see Dotson, 2014)? Pohlhaus (2017, p. 13) identifies three intertwined, distinctively epistemic aspects: first, epistemic oppression corrupts the knowledge of certain individuals by preventing them from acquiring knowledge and/or by suppressing their testimony of already acquired knowledge. Second, it leads to dysfunctional epistemic practices, insofar as it distorts or thwarts understanding of certain facts or experiences. Third, epistemic oppression is structurally inscribed in certain epistemic institutions. It is enacted not by any (non‐epistemic) social, political, or institutional actors, but rather in structural practices of ignoring, distorting, or discrediting certain intellectual traditions and knowledge cultures.^21^ Ultimately, these structural practices undermine themselves as reliable generators of knowledge and lead to a mere reproduction of ignorance regarding the mechanisms of oppression in question. Thus, epistemic oppression impairs the knowledge production and acquisition regarding certain issues, in ways that harm mostly, but not only, the oppressed themselves (cf. Pohlhaus, 2017, p. 14).

So much for a general characterization of epistemic injustice and its oppressive and epistemic aspects. But what exactly is epistemic *exploitation*?^22^ Epistemic exploitation occurs in discursive situations in which we have structural (e.g., socioculturally, economically, ethnically or racially grounded) and unjust power imbalances between participants. Speaker and addressee roles are not prefixed and are interchangeable; the discursive‐epistemic inequalities between the participants remain, however, preserved. On the one hand, we have participants representing and appropriating a majority discourse with the respective epistemic resources, let us call them speakers A. On the other hand, we have minoritarian speakers B. Whereas A are supposedly ideal, B are considered “substandard” epistemic agents.

Now, epistemic exploitation is present whenever A exert, reinforce or maintain epistemic oppression against B by engaging in the following discursive behavior: A exert pressure on B to provide A with information about the nature of B's oppression. The pressure involves B having no alternative to providing unacknowledged or uncompensated and emotionally draining work to educate A about their (B's) own oppression. The obligation to educate, then, is not autonomously chosen by B but implicitly or explicitly imposed by A. This load further depletes B's epistemic and emotional capacities than they already are due to the prevailing cultural, socioeconomic, racial, etc. inequalities in which epistemic exploitation is always embedded. Another effect of what Berenstain construes as the “double‐bind” of epistemic exploitation is that B are prevented from making better use of their knowledge about their own exploitation and vulnerability. Instead of using their knowledge to educate their own or other marginalized groups and bring about social change in their favor, they are forced to make this knowledge available for the enlightenment of their oppressors. And the oppressors do not just get this epistemic work gratis, it is additionally taxed with the emotional labor of the oppressed.^23^

Some clarifications on the nature and mechanisms of exploitation are needed: First, it should be noted that the pressure on B need not be exerted directly by A for B to feel obliged to provide educational labor. The pressure is typically structural and internalized. Moreover, the above‐mentioned double bind entails that the oppressed have no real choice but to yield to the pressure. As Berenstain shows (Berenstain, 2016, esp. 576), the privileged ones feel entitled to exploit the cognitive and emotional energy of the marginalized for their own profit. In the face of this entitlement, the marginalized fear reproach or further exclusion if they do not meet the expectations of the privileged, for example, if they refuse to answer questions, pose skeptical counter‐questions or show intense emotional reactions in a discourse that is supposed to be emotion‐free and purely rational. The marginalized cannot but take part in the game; if they refuse to play along, they risk further damage to their reputation as rational participants in the discourse.

The information that the marginalized are supposed to provide can be manifold: statistical evidence, argumentative or descriptive explanations, educational material, testimonials, and so forth. They ought to corroborate the fact that the oppressed are oppressed or substantiate their personal experiences of oppression. This is crucial for understanding the unjust relationship between the parties, and the profit and perpetuation of exploitation involved. It's worth repeating the point, for it is rather extraordinary what happens here: The dominant group, which benefits from the oppression already anyway—demands enlightenment from the oppressed about the nature of oppression itself, thereby further draining the epistemic and emotional resources of the oppressed and perpetuating exploitation. As Berenstain pointedly puts it, a central feature of epistemic exploitation is “to keep the oppressed busy doing the oppressor's work” (Berenstain, 2016, p. 574).

The oppressors employ different discursive strategies. A common one consists of rhetorical camouflages, whose goal is to transvalue the exploitation as an epistemic virtue. Note here how this is parallel with the obfuscation tactics of the bullshitter, who seeks to cover up his activity. The euphemistic distortions and trivializations become linguistically manifest in expressions of intellectual curiosity (“I'm just curious.”), alleged benevolence (“I'm only asking to help”). A strategy that often goes along with this is to mask epistemic exploitation as a reliable or even indispensable method for the establishment of truth. The speakers profess methodical neutrality or rigor (“Without getting to the bottom of this, we can't find a solution.”) or critical inquiries and objections, which rather serve to unsettle the exploited than to clarify the problems at stake, are presented as commonly accepted rhetorical patterns of rational discussion (“I am only playing the devil's advocate.”), or one offers so‐called “alternative explanations” (“With a bit of goodwill one can see it quite differently.”) (see Berenstain, 2016, pp. 571–572).

Such trivializing strategies of concealing epistemic exploitation of course do not stop short at the rhetorical level. They go hand in hand with questioning, rejecting and ignoring the knowledge conveyed by the marginalized. Two fundamental, interrelated processes should be highlighted here (see Fricker, 2007).^24^ First, the epistemic discrediting known as testimonial injustice: a skepticism regarding the credibility of certain discourse participants or the general validity of what they report, (mis)taken to be purely subjective experiences or isolated incidents (“But this is only an individual case, however abominable it may be, I just can't imagine that your experience generalizes.”). Secondly, epistemic exploitation involves the unjust selection, misappropriation or distortion of certain epistemic resources or a form of so‐called “hermeneutic injustice.” It occurs when the discursive‐epistemic infrastructure (court hearings, classroom or plenary discussions, teaching syllabi, newsroom‐hierarchies, etc.) is constructed in a way that it only considers or intentionally favors the interests of the dominant participants. The skewed epistemic infrastructure creates systematic, so‐called “hermeneutic lacuna” between majority and minority speakers. These gaps prevent the majority group, and often the marginalized themselves, to adequately understand the social experiences of the latter. But hermeneutic injustice can also assume more active and drastic forms of misappropriation and discursive manipulation, either in terms of so‐called “willful” (Pohlhaus, 2012) or “contributory” (Dotson, 2012) hermeneutic injustice. This involves ignoring commonly and readily available epistemic resources, evidence, and so forth, or using them in biased or manipulative ways such that the epistemic performance of the marginalized becomes deliberately compromised. Typically, hermeneutic injustice is also accompanied by forms of discursive silencing, and notably by “testimonial quieting” (Dotson, 2011), whereby the marginalized are not identified as knowers at all. Or they result in the self‐censorship referred to by Eddo‐Lodge, which has also been characterized as a “testimonial smothering” (Dotson, 2011), whereby marginalized trim their own testimonials to immunize themselves against further injustice and exploitation.^25^

## 4 EPISTEMICALLY EXPLOITATIVE BULLSHIT: THE SARTREAN ACCOUNT

We now have an idea of what bullshit and epistemic exploitation are. In this section, I show that Sartre's analysis of anti‐Semitic discourse presents a sketchy but highly original, integrative conception of these two phenomena. As dozens of passages in his essay *The Anti‐Semite and the Jew* demonstrate, Sartre conceives of the anti‐Semite as both at the same time: an epistemically exploiting bullshitter. As already hinted at, such a discursive “bastard” is more dangerous than an ordinary, isolated bullshitter since he exploits power relations beyond the epistemic‐discursive domain and hence deeply disrupts the fabric of social and political interaction. But he is also more destructive than the epistemic exploiter simpliciter, for EEB is not only linked to destructive political agency but also to a complete debasement of the value of truth and rational discourse. This invalidation goes not only beyond the bullshitter's alethic insouciance, but it is also more explicitly antagonistic than epistemic exploiters' strategies of marginalization, which are often the result of implicit biases or other engrained and unreflective structural injustices.^26^

Although EEB can neither be reduced to bullshit nor to epistemic exploitation but represents an irreducible form of speech, I will proceed analytically and first compare EEB to bullshit, then to epistemic exploitation, showing along the way what EEB shares and what it distinguishes from both.

### 4.1 EEB and bullshit

Let us start by considering the relation between EEB and bullshit. Recall that bullshit is defined by two characteristics: first, by the generalized alethic insouciance, or the fundamental unconcern for the very difference between truth and falsity; second, by a distinctive intention to deceive, which does not aim at concealing the falsity of statements (as in conventional lies), but at concealing the speaker's alethic insouciance itself. As already evident from the quote in the motto of this paper, Sartre (1995b[1946], pp. 19–20) explicitly points to these two aspects. We read there, for instance, about the “absurdity” of the anti‐Semite's speech, of which he is, to be sure, not only fully aware but which he frivolously endorses, as he considers himself to have “the right to play with discourse” and gives “ridiculous reasons” for his knowingly unjustified beliefs. As Sartre also implies immediately before this passage, it would be accordingly “futile and frivolous” to demand from the anti‐Semite better reasons for his beliefs, for “he has placed himself on other grounds from the beginning”, grounds that have nothing to do with rational discourse, where one ought to provide as good reason as possible for one's beliefs. Moreover, as Sartre later puts it, “it is of no importance” to the anti‐Semite if his beliefs or judgments about Jews might turn out as “erroneous” or only being prejudiced, for he does not feel any obligation to see what is true or false in them. It is “the Jew [who] must decide for himself whether [the anti‐Semite's judgments are] true or false” (Sartre, 1995b[1946], p. 82).^27^

But the anti‐Semite is not only unconcerned with whether his “arguments” are “sound” or “persuade” anybody, insofar as he does not care about their justification and in fact about the truth or falsity of his beliefs altogether; moreover, he aims at misleading his interlocutors (the Jews and the philo‐Semites) or to “disorient”^28^ them (Sartre, 1995b[1946], p. 20).^29^ On top of that, he enjoys being insincere or “delights in acting” in what Sartre calls “bad faith” (Sartre, 1995b[1946]). Sartre's bullshitter, then, clearly fulfills also the second condition of bullshit, namely a deception regarding his subversion of rational discussion and his alethic insouciance. But Sartre is far more nuanced than Frankfurt and the contemporary bullshit debate as to the nature of this deception. According to Sartre, what is at stake here is only indirectly a deception of *others*; primarily, it is a specific form of *self*‐deception, namely the self‐deceptive insincerity that he famously discussed in terms of bad faith (*mauvaise fois*).

I will argue that this particular form of insincerity is a central feature of the Sartrean conception of EEB, and indeed grounds, both epistemologically and existential‐ontologically, the discourse and the very being of an epistemically exploitative bullshitter.^30^ Largely abstracting from the idiosyncratic Sartrean framework, I want to suggest that we get a clearer understanding of EEB if we consider how bad faith relates to the bullshitter's alethic insouciance (Section 4.1.1), and how it involves the complex socio‐dynamic mechanisms of epistemic exploitation, which I elaborate as a prejudiced group‐identification (Section 4.2.1), as well as the ensuing collective diffusion and forced delegation of epistemic responsibility (Section 4.2.2).

#### 4.1.1 Bullshit and (bad) faith

Bad faith, for Sartre, is a fundamental existential attitude of self‐negation and self‐reification. In the broadest terms, it is a self‐deceptive negation of the fact that one is always situated in a certain way but never fixed to just one particular situation. Moreover, bad faith involves a negative identification, or reification, of oneself: One identifies one's spontaneity in determining one's human reality with the factual and unchangeable reality of objects. In other words, one negates, or flees, one's ever‐open horizon of possibilities, the freedom to choose oneself, or to shape one's situatedness. This is the existential‐ontological gist of bad faith. But as the very term indicates, and as should be emphasized in our context, bad faith is not just an existential‐ontological structure of human reality—it is also a distinctively epistemic phenomenon. As Sartre explicitly remarks, “the essential problem of bad faith is a problem of believing” (Sartre, 1995a[1943], p. 67).^31^

From the epistemological perspective, bad faith is a negative doxastic attitude towards one's beliefs (primarily but not solely about oneself). Indeed, the best way to approach the epistemological nature of bad faith is by insincerely distinguishing between believing something (truths or falsities) and intending to deceive others such that *they* believe falsities. But one must not only be wary about modeling bad faith on conventional lies directed at others; one must be just as careful not to understand bad faith simply in terms of *lying to oneself*. To be sure, Sartre acknowledges that both in lying to oneself or others and in cases of bad faith one “is hiding a displeasing truth or presenting as truth a pleasing untruth” (Sartre, 1995a[1943], p. 49); but the obvious difference between an ordinary lie and bad faith is that in bad faith there is “no duality of the deceiver and the deceived” (Sartre, 1995a[1943]). Rather, the subject in bad faith must present to herself the content of the belief as untrue, and yet believe in that (false) content, all the while having a grasp of the content of the belief as well as of what she is doing. Hence, Sartre is confronted with an apparent paradox, which mirrors the one that has been extensively discussed in the literature on the possibility of intentional self‐deception. I cannot engage with this discussion here nor elaborate on Sartre's own resolution, which draws on the peculiar interplay between pre‐reflective and reflective (self‐)consciousness. Let me just emphasize those points in Sartre's account that are relevant for our prime target here, the bullshitter and his similarly peculiar deception.

First, bad faith is not a momentary instance of self‐deception, nor is it a static or state‐like belief. Moreover, one does not passively “undergo” one's own bad faith or “is infected with” it. Rather it is an intentional engagement with oneself, or a “project” (*projet*). Part of Sartre's resolution of the apparent paradox of self‐deception is based on this project‐character of bad faith. Thus, the “project of bad faith […] implies a comprehension of bad faith as such and a pre‐reflective apprehension (of) consciousness as affecting itself with bad faith” (Sartre, 1995a[1943], p. 49). In a certain sense and on a certain level of consciousness, then, the person “in” bad faith has the deliberate aim of infecting herself with untruth (about oneself). The intricacies of this rather miraculous epistemic dialectic need not concern us. What is important for our purposes is its similarity to bullshit: analogously to the project‐character of bad faith, Frankfurt characterizes the bullshitter's intentional deception as something that concerns her “enterprise,” rather than any of his particular statements; the bullshitter “misrepresents what he is up to” (Frankfurt 1986, p. 54)—not so much what he is saying.

Secondly, and more importantly, Sartre determines the intentional project‐character of insincerity in two further respects: bad faith as *mere faith* and as itself based on bad faith, or as “a decision in bad faith about the nature of faith” (Sartre, 1995a[1943], p. 68). Herein lie the analogies to the bullshitter's knowingly insufficient justification for his beliefs and his generalized alethic insouciance.

For one, bad faith is *bad* faith because it is, wittingly, *mere faith*. It is not merely unjustified or insufficiently justified belief, but belief where the believer *knows* that the justification is insufficient and yet holds on to it, pretending or presuming that it equals knowledge. “[Bad faith] stands forth in the firm resolution not to demand too much, to count itself satisfied when it is barely persuaded, to force itself in decisions to adhere to uncertain truths.” (Sartre, 1995a[1943]).

Bad faith as *mere faith*, or knowingly unjustified belief, mirrors Stokke's and Fallis's construal of bullshit: it is marked by a decision to remain indifferent as to whether one's belief is in any relevant justificatory way grounded in the evidence available to one. Accordingly, Sartre opposes (bad) faith in this sense at one point to “science” and its “searching for evidence” (Sartre, 1995a[1943], p. 70). But such faith is faulty in more than just its lack of evidence or insufficient justification. It is marked by a motivated faultiness. Thus, the acquisition of such faith, rather than just the result of factually insufficient or faulty justification, is the result of an epistemic bias resembling so‐called confirmation bias: Following his interest to confirm certain beliefs, the believer in (bad) faith sets up his own evidentiary standards, standards that are known to him to be too low to establish reliable, even if defensible, belief (see also Detmer, 2014, pp. 126–127). Consider again the ardent conservative, who will require higher standards of fact‐checking, historical or statistical coherence, and so forth for a CNN compared to a Fox News report. But this is still an epistemologically *thin* version of bad faith since it keeps in place the distinction between *all‐things‐considered* justified or unjustified, and between true or false beliefs.

But Sartre also provides an epistemologically *thicker* notion of bad faith, when he claims that bad faith is a “decision” about and affects “the nature of truth” itself, a decision to “not [hold] the norms and criteria of truth”. This also involves a more radical epistemic strategy than to just be content with “*non‐persuasive* evidence” and to “adhere to uncertain truths” (1995a[1943], p. 68). Rather, it involves precisely the *generalized* alethic insouciance that also characterizes bullshit. According to this more radical understanding, bad faith amounts to a “self‐destruction of belief” (1995a[1943], p. 70). It corrupts one's ability, and indeed the very possibility, to draw a clear line between true and false beliefs. As Sartre aptly remarks, with the decision to be insincere in the mode of bad faith, it becomes ultimately “indifferent whether one is in good or in bad faith because bad faith reapprehends good faith” (1995a[1943], p. 70, Fn. 9)—a remark once again reminiscent of the generalized alethic insouciance of the bullshitter.

Indeed, we can best capture the *epistemic* dimension of Sartrean bad faith on the basis of our understanding of bullshit as generalized alethic insouciance. Bad faith is a negative attitude not so much towards particular beliefs or a self‐deception regarding some particular (existential or other) facts, but a destructive attitude towards the nature of beliefs (about oneself, others and the world). Beliefs can only be formed and held if one has faith in their potential truth, and this faith is corrupted by bad faith. Bad faith, then, is not lying to oneself *simpliciter*, but lying about one's (false) relation to the nature of truth as such. In Sartre's epistemological notion of bad faith, we can clearly see then that the violation of both Williams' accuracy and sincerity condition by the bullshitter are but two sides of one and the same epistemic vice.

### 4.2 EEB and epistemic exploitation

So much for the analogies and differences between Frankfurt's and Sartre's bullshit‐conceptions, and the epistemological role of bad faith in the latter. I now turn to the relationship between EEB and epistemic exploitation (before I come back to the existential‐ontological dimension of bad faith and its social‐psychological implications for epistemic injustice).

The above motto provides us again with the initial clues. There we read of the “obligation” imposed on the Jew or the philo‐Semite to abide by the rules of rational discourse, while the anti‐Semite does not feel bound by these rules, but rather considers himself in the “right” to “play” with discourse in a bullshit fashion. Further on, Sartre specifies the obligation imposed on the marginalized to educate majority speakers about the nature of their oppression, and the concomitant burden that can be characterized as the “emotional hostage‐taking” of epistemic exploitation: “The perpetual obligation to prove that he is French puts the Jew in a situation of guilt. If on every occasion he does not do more than everybody else, much more than anybody else, he is guilty” (Sartre, 1995b[1946], p. 87). This indissoluble double bind of (self‐)imposed obligations is also bluntly expressed by Sartre when he states: “the situation of the Jew is such that everything he does turns against him” (1995b[1946], p. 141).

Sartre also points to an aspect that is related to emotional hostage‐taking and which is crucial to understand the power dynamics in epistemic oppression but is less marked in the contemporary literature, namely, an involuntary solidarity with one's own oppressed group. With Sartre, we can see that there is a direct connection between the imposed duty of the oppressed to provide epistemic evidence or explanations for their oppression and the heterodox self‐obligation to solidarize with one's own exploited group. The oppressed have a forced co‐responsibility for the words, deeds, supposed or real mistakes of their peers. Sartre gives a telling example that can readily be applied to the epistemic‐discursive domain: “In the case of war and civil disturbance, the ‘true’ Frenchman has no proofs to make. He simply fulfills his military or civil obligations. But it is not the same for the Jew. He may be sure that people are going to make a strict count of the number of Jews in the army. Thus, he suddenly finds himself answerable for all his co‐religionists.” (1995b[1946], pp. 85–86).

Regarding another defining characteristic of epistemic exploitation—testimonial injustice—Sartre is equally illuminative. As we learn again from the quote in the motto, the anti‐Semite “discredits” his discursive victim by adducing “ridiculous” reasons (which is also reminiscent of the strategy of bald‐faced liars). According to Sartre, there is an essential connection in anti‐Semitic discourse between the alethic insouciance of bullshitting and epistemic exploitation, and it is this connection that makes EEB heftier than a mere frivolous playing with truth.

One of the central points of the Sartrean account of EEB, then, is to show that the anti‐Semite does not play with truth and discourse for the sake of playing, but because he aims to discredit his opponent and, in fact, feels entitled to epistemically exploit him. The anti‐Semite à la Sartre thus goes far beyond a merely cynical form of discrediting that we can witness in bullshit. And he is not only “impervious to reason and to experience,” ignoring available evidence and paying no respect to shared conventions of discourse (which amounts to hermeneutic injustice); moreover, he casts doubt on or outrightly rejects unexamined evidence that might contradict him.

At a crucial juncture, where the strategic‐discursive threads of bullshit inextricably converge with those of epistemic exploitation (and which I have already quoted in excerpts), Sartre writes:

But it is of no importance that [the anti‐Semite has] an erroneous notion; the fact is that it is a group error. The Jew must decide for himself whether it is true or false; indeed he must prove it. And yet people will always reject the proof which he furnishes. […] In vain may he argue about his culture, his accomplishments; it is Jewish culture; they are Jewish accomplishments. He is a Jew precisely in that he does not even suspect what ought to be understood. Thus an attempt is made to persuade him that the true sense of things [*genuine* France, *genuine* values, its *genuine* morality] must always escape him […] (Sartre, 1995b[1946], p. 82)

The anti‐Semite, based solely on the negative stereotypes regarding his interlocutor, questions the credibility of what he hears. The Jew is met “always with a distrust that drives him on each occasion to ‘prove himself’.” (Sartre, 1995b[1946], p. 85) The skepticism is similar but goes beyond the unfair and biased epistemic strategy involved in the bullshit‐like faith mentioned above, whereby one demands a higher standard of evidence and justification from certain others than oneself, depending on one's epistemic interests. It rather resonates with testimonial and hermeneutic injustice involved in epistemic exploitation.

But the skepticism Sartre describes is even more comprehensive than the standard cases found in the epistemic injustice literature and has a different hermeneutic angle: For Sartre, it involves, in one big sweep, the epistemic, aesthetic, emotional and moral psychological capacity of the marginalized to grasp any supposedly “deep” or “true” facts concerning the issues under consideration—truths that are hidden not only for the non‐initiated but in principle escape the understanding of the marginalized. Such a delimitation of the bounds of the knowable and affectively, aesthetically or moral‐psychologically graspable is a crucial mechanism of EEB, which again has largely escaped the spotlight of contemporary work on epistemic injustice. What happens here cannot be reduced to hermeneutic injustice, testimonial smothering or anticipatory epistemic injustice. What happens is a discursive ingroup/outgroup‐demarcation, whereby the interlocutors are pre‐assigned to distinct, and opposed, epistemic groups—here: “us,” who know and feel appropriately or authentically about a domain under consideration, there: “them”, who in principle cannot thus know or feel.

#### 4.2.1 Bad faith as aletheic ur‐anxiety and “identity‐prejudicial stereotyping”

To substantiate the Sartrean account of testimonial and hermeneutic injustice and its sociopsychological dynamic, we need to return to Sartre's account of bad faith. For it is bad faith that explains what *motivates* the mentioned radical skepticism in EEB. Moreover, following Sartre, it is the reification and essentialization of human facticity and the rejection of epistemic responsibility for the truth about one's own and others' natures in bad faith that leads to epistemic injustice and, as we shall see in the next section, to the collective exploitation of the marginalized speakers.

So, why does the anti‐Semite, according to Sartre, lull himself into bad faith? Surely, there must be more to his decision for bad faith than merely to conceal his alethic insouciance. Although Sartre does not explicitly list the reasons in his *Anti‐Semitism*‐essay,^32^ we can distill the following argument: First, the anti‐Semite reifies his own and the character of the Jew as some allegedly pure essence: here the “true” Frenchman, there “*the* Jew,” with all his stereotypical, unchangeable characteristics, which he shall never escape. The reason for this existential‐ontological hypostasis, in turn, lies in the fear of the spontaneity, situatedness, and freedom of human existence, which the person in bad faith “flees.” But this existential fear has again an *epistemological* dimension: the flight from responsibility for the value and burden of truth, or what I call “alethic ur‐anxiety.”

In alethic ur‐anxiety, the usual correlation between subjectively felt epistemic power and validity of one's beliefs, on the one hand, and one's sensitivity to evidence and justification, on the other, is broken. The anti‐Semite does not hold on to his beliefs because he considers them justified. But neither does he close his mind to rational and empirical evidence that might contradict his stereotypical views because he has rock‐solid beliefs, however stereotypical they may be: “If […] the anti‐Semite is impervious to reason and to experience, it is not because his conviction is strong. Rather his conviction is strong because he has chosen first of all to be impervious” (Sartre, 1995b[1946], p. 20). And this radical closed‐mindedness is grounded in bad faith vis‐à‐vis the (transcendent) nature of truth and the structure of the inauthentic self‐and other‐relation: The anti‐Semite “is attracted by the ‘durability of a stone.’ […] We have here a basic fear of oneself and of truth. What frightens [him] is not the content of truth, but the form itself of truth, that thing of indefinite approximation” (Sartre, 1995b[1946], pp. 18–19).^33^

At this point, then, the epistemological and existential‐ontological function of bad faith coalesce: The flight from assuming responsibility for one's ever‐indeterminate character just mirrors the flight from the responsibility to provide epistemic justification for one's beliefs and for the never‐ending quest for certainty. Indeed, I argue that the essentialization or reification of oneself and the essentialization of truth (about oneself, the world and others) are just two sides of the same coin, namely the alethic ur‐anxiety in bad faith, as sketched out in Sartre's *Anti‐Semitism* essay.

Now, importantly, bad faith does not just involve *self*‐reification; as Sartre insists in the Anti‐Semitism essay, it also involves the reification of *others*. In fact, the two are essentially correlated. The subject of bad faith reifies himself as belonging to an essentialized (here: racialized) “us,” while attributing a fixed essence to those excluded from one's ingroup.^34^

This dialectic of self‐and other‐stereotyping nicely ties in with Fricker's (2007) notion of “negative identity‐prejudicial stereotype” and how it works in testimonial injustice. Fricker defines “identity prejudice” as a “prejudice against people qua social type” and characterizes the stereotype as a “disparaging association between a social group and one or more attributes, where this association embodies a generalization that displays some (typically, epistemically culpable) resistance to counter‐evidence“. Fricker contends that this is the fundamental mechanism underlying testimonial injustice. The idea is that this prejudiced stereotype “distorts” from the outset “the hearer's perception of the speaker” and his “credibility judgment,” such that hearer cannot but perceive the speaker as untrustworthy (Fricker, 2007, pp. 35–35). Applied to our context, we can thus see how the anti‐Semite, based on his prejudiced essentialization, cannot perceive Jews or philo‐Semites as anything but a lying pack.

#### 4.2.2 EEB and the collective diffusion of epistemic responsibility

By employing the basic insights of Sartrean bad faith, we get a better grip on how the flight from the burdens of epistemic accuracy and from the responsibility for the value of truth motivates EEB. Moreover, we can see how the reification and essentialization involved in bad faith mirror the identity prejudice in testimonial injustice. But the sociopsychological dynamic in prejudiced stereotyping leads us directly to a related one, which Sartre again brings into sharper relief than most contemporary work on epistemic injustice (including notably Fricker's). As I want to show now, bad faith also involves a *collective* diffusion of epistemic responsibility, which underlies the unjust and exploitative dimension of EEB^35^: in fleeing one's spontaneity, the epistemic exploiter does not just group‐identify with his fellow‐travelers and reifies himself as belonging to an essentialized “us”^36^; moreover, he externalizes his personal epistemic responsibility to the dominant discourse and then collectively delegates the responsibility for obtaining adequate information to his victims, not so much out of aletheic fear than to perpetuate oppression.

The portrait of the epistemically exploitative bullshitter would be incomplete in an essential respect if we viewed him in social isolation. EEB is not an epistemic vice of individuals or an individual offense. Sartre contends that the typical anti‐Semite is a “man who fears every kind of solitariness” and “is a man of the crowd.” But this not only entails that “there is no example of an anti‐Semite claiming individual superiority over the Jews” (1995b[1946], p. 22; see also pp. 27–30). Sartre's claim is more nuanced. For Sartre, anti‐Semitic discourse is an essentially *collective* project. To appreciate this claim, one need not share Sartre's radically social‐constructivist thesis regarding the nature of “being Jewish” or what makes someone a Jew or a member of any other ethnicity, religion, etc. (though I have certain sympathies for it). Sartre notoriously holds that what is (only) “common” to Jews is “the situation of a Jew,” which is to “live in a community which takes them for Jews” (1995b[1946], p. 67). In other words, “the Jew is one whom other men consider a Jew” and thus “it is the anti‐Semite who *makes* the Jew” (69). But even if one does not subscribe to these controversial claims,^37^ Sartre's insistence on the essential collectivity of EEB, as I take it, is justified. Indeed, in my reading, not only does Sartre highlight the underappreciated collective aspects of many instances of bullshitting. Sartre also succeeds better than most contemporary writers also in integrating the interpersonal and institutional aspects of epistemic oppression, and in showing how they typically go hand in hand.^38^

Specifically, Sartre accentuates two interrelated aspects of the collectivity involved in EEB: One has to do with the alethic insouciance of the bullshitter, the other with the asymmetrical power structure of oppression. We have already seen that erroneous beliefs do not bother the anti‐Semite, not just because of his alethic insouciance but also because and insofar as they are “group errors” (1995b[1946], p. 82). Thus, the Sartrean bullshitter relieves himself from any pressure to answer for his errors or to justify his alethic unconcern on his own and delegates the epistemic responsibility to his fellow‐travelers. Instead, he externalizes his own personal epistemic responsibility and relies on the shared epistemic practices and resources of his ingroup. However, these practices and resources are knowingly skewed. They exclude dissenting voices or diverging perspectives from the outset and do not even strive for approximating truth. The exploitative bullshitter thus frees himself from any commitment to the truthfulness of the discourse and the virtue of truth. Moreover, and this is an equally crucial step in the process of diffusing epistemic responsibility, the exploitative bullshitters *collectively* delegate the epistemic responsibility to their victims.

In this social‐psychological dynamic, EEB then perfectly and noxiously blends the *untruthful* and the *oppressive* dimension of the discourse. In enacting his untruthful speech, the exploitative bullshitter cements asymmetrical power structures, and “affirms” his sense of belonging “to the elite” (in this case “an aristocracy of birth”) (Sartre, 1995b[1946], p. 27).^39^ Moreover, the group of the oppressors and the oppressed are performatively co‐constituted in the speech acts uttered (or shouted), “in chorus”^40^: “He has made himself an anti‐Semite because that is something one cannot be alone. The phrase, ‘I hate the Jews,’ is one that is uttered in chorus; in pronouncing it, one attaches himself to a tradition and to a community” (Sartre, 1995b[1946], p. 22).

EEB thus proves to be not a solitary discourse, but a kind of choral speech, or a collective performative act. Of course, as already indicated, there is more at stake than just the power of performative speech acts or the sovereignty over merely epistemic terrain. What's at stake is ultimately the political question of power and exclusion: “[The] anti‐Semite […] wishes to be the disciplined member of an undisciplined group; he adores order, but a *social* order. […] he wishes to provoke political disorder in order to restore social order […] one from which Jews are excluded.” (Sartre, 1995b[1946], p. 32).

But is there not still more at stake? I shall now address an obvious objection to the effect that—notwithstanding the charges of bullshitting, deceiving and exploiting— we have so far treated the (Sartrean) anti‐Semite with kid gloves. Thus, the critic might note that anti‐Semitism is not merely a discursive and epistemic phenomenon, however political that may be, but a form of political violence with very real psychological, social, or physical consequences for the victims, and also for the anti‐Semites themselves. Nothing makes this clearer than the Shoah, the immediate historical context of Sartre's essay, which I have entirely disregarded. To be sure, Sartre emphasizes the aspect of political violence again and again, for instance when he writes that the anti‐Semite not only is impervious to reason but “has chosen also to be terrifying” (1995b[1946], p. 20), “has chosen to be a criminal,” (1995b[1946], p. 50) and indeed “[has chosen] nothing save the fear he inspires in others” (1995b[1946], p. 21). Moreover, Sartre repeatedly describes the affective dimension of anti‐Semitism as a passionate hatred (e.g., 1995b[1946], pp. 19, 22, 28, 50), and warns that the anti‐Semite must not be provoked for “no one knows to what lengths the aberrations of his passion will carry him” (1995b[1946], p. 21). Finally, the anti‐Semite is not only one who gives speeches or produces EEB, but “a hooligan,” who “beats up,” “purges” and “robs” (1995b[1946], p. 33), to put it still mildly against the foil of the Shoah. Conversely, the Jew is not only obliged to furnish epistemic proofs for his case and must not only “prove himself” in words but also in deeds, in civil, political, and military life.

I certainly do not want to level the distinction between the discursive‐epistemic aspects of anti‐Semitic or racist agitation and those aspects that go beyond these and involve psychological or physical violence.^41^ But I think that these differences matter more for understanding the factual course and effects of the phenomena than the nature or the genesis of the underlying processes. And as we know all too well from history and recent political developments, discursive and epistemic perversion is typically the first decisive step towards antagonism that involves not only speech and counter‐speech but eventually violence and counter‐violence. Thus, we also learn from Sartre that we can only adequately understand and counter hate speech, for example, if we understand how it feeds on bullshit and epistemic exploitation, whose combination brews the toxic mixture.

## 5 CONCLUSION

At the beginning of this paper, I remarked that truth and truthfulness are not just epistemic phenomena but have a genuinely political dimension. As Balibar aptly notes, the “struggle for knowledge” is ultimately a “political practice” insofar as the communicative negotiation of truth is “structured by relationships of ignorance and knowledge, superstition, and ideological antagonism” (Balibar, 1985[2008], p. 98). As we have seen, these relationships are partly determined by strategic forms of subversion of sincere communication: Notably by the generalized alethic insouciance and its deceptive disguise in bullshit as well as by the mechanisms of epistemic exploitation.

However, the explanation of the subversion of sincere communication we find in the two epistemological debates discussed here both fall short, and in particular insofar as they proceed in isolation from each other, as they in fact have to date. Thus, the debate about bullshit (and bullshit‐like forms of non‐alethic speech) remains too closely oriented to the model of interpersonal lying. Consequently, much of the debate is limited to the question of whether speakers can deceive others about the truth of their assertions without actually lying, or whether an intention to deceive an addressee is a necessary requirement for all forms of lying. What is ignored here is that all politically interesting forms of non‐alethic speech are embedded in broader socio‐communicative contexts. What is often at stake is not just the communication of truth/falsity, but the epistemic subordination of others and the strategically unequal distribution of access to information or evidence.

While the critical epistemology literature on epistemic injustice and, in particular, on epistemic exploitation address this very point, it has its own blind spots: First, it ignores the fact that epistemically unjust discourse often involves precisely bullshit or bullshit‐like forms of speech to undermine and discredit the epistemic contribution of minority speakers; second, this literature lacks any detailed analysis of the self‐deceptive or insincere dimension of the rhetorical strategies of camouflage, concealment or trivialization involved in epistemic exploitation.

In contrast, I have attempted to demonstrate that Sartre's analysis of anti‐Semitic discourse takes all these dimensions into account and shows how they are essentially connected. The Sartrean account thus captures a form of speech that can neither be reduced to bullshit (or other non‐alethic speech) nor to epistemic exploitation, but only captured through their interplay. Moreover, it brings into sharper focus the sociopsychological dynamics in the delegation and collective diffusion of epistemic responsibility that play a key role in epistemic exploitation. And this, I have suggested, has to do with Sartre's original conception of self‐deceptive insincerity or the “alethic ur‐anxiety” in bad faith.

Ultimately then, the political negotiation of truth and the political struggle for knowledge is structured not only by the relation of ignorance, knowledge, and ideological antagonism, but just as much by the relation of deceiving oneself and others, or of sincerity and insincerity towards oneself and others. As we learn from Sartre, alethic insouciance, insincere or self‐deceptive belief and epistemic exploitation are different but irrevocably intertwined aspects of the same antagonistic discourse.^42^ The *sui generis* type of discourse is what I have coined “epistemically exploitative bullshit.” Judged alone by its almost tantalizing, discursive‐epistemological complexity and its inherent instability—which it inherits from the moral‐psychological instability of self‐deception—one might presume that is a very rare phenomenon. But for better or worse, I suspect that the opposite is rather the case.

## Endnotes

^1^
As we shall see, the latter (saying what one believes to be false) does not without further qualifications amount to lying.

^2^
One of the few exceptions represents the trenchant analyses in Breeur (2019); see also Cassam (2021).

^3^
Accordingly, the following caveat should be noted: I will present an unabashedly selective reading of the nature of anti‐Semitism in Sartre's work, focusing only on those aspects of Sartre's conception of anti‐Semitic speech that are relevant to the contemporary discussions of bullshit and epistemic injustice. Given the intriguing nature of Sartre's impact on these discussions, this seems justified. For further caveats regarding Sartre's controversial conception of the Judaism, see below, Fn. 28. Finally, I will not discuss Sartre's longstanding critical engagement with racism and (post‐) colonialism either (see, e.g., Sartre, 1988[1948], 1983), nor the relevant mutual influences between Fanon and Sartre, even if these would be worthwhile for a broader understanding of Sartre on epistemic exploitation; see more on in Alcoff (2005); Gloag (2020); Gordon (1995); Van Leeuwen (2007, 2008). Regarding Fanon, let me just mention that some of his remarks in *Black Skin, White Masks* closely resonate with the Sartrean reconstruction of bullshit I present in this paper, for instance when he writes: “In an age of skepticism when, according to a group of *salauds*, sense can no longer be distinguished from non‐sense, it becomes arduous to descend to a level where the categories of sense and non‐sense are not yet in use.” (Fanon, 1952[2008], xiii).

^4^
But also—and more markedly than it has been so far—from a variety of cognate non‐conventional forms of mendacity and sham, ranging from various bald‐faced lies to disinformation; I cannot endeavor that here, but see the notes 5, 6 and 17.

^5^
By “conventional” I mean those lies that fulfill all four conditions below. In attempts to sever the connection between lying and the deception condition (esp. by Carson, 2010; Fallis, 2009; Sorensen, 2007), see Stokke (2013; 2018) and critically Lackey (2013) and the notes 6 and 12 below.

^6^
Today, we have a cornucopia of bullshit‐conceptions, which emerged from critiques of Frankfurt's initial proposal. However, most of these just represent a watering down of the original conception to the point of unrecognizability (cf. also Saul, 2012, chap. 1). Some post‐Frankfurtian conceptions, for instance so‐called “evasive” (Carson, 2010, pp. 60–61) or “bald‐faced” bullshit (Fallis, 2015b), are virtually indistinguishable from other forms of bald‐faced lies. Bald‐faced lies are lies where the intention to deceive is not fulfilled and the falsity of the speaker's assertion is common knowledge, but where the speaker aims to strategically disrupt the epistemic capacity of the addressee, for example, by undermining her ability to correct the obviously false statement (“acknowledgment lies”) or by preventing that she gains knowledge concerning the statement in question (“knowledge lies”); see Sorensen (2010). But, *pace* Sorensen (2011), Frankfurtian bullshit must also be distinguished from so‐called “misspeaking,” or a form of insincere guessing. Yet others miss the distinctiveness of Frankfurt's conception by reconstruing bullshit as “obscure” (Cohen, 2002) or “incomprehensible speech” (Ivanković, 2016). On what exactly is distinctive of Frankfurtian bullshit compared to all these non‐alethic forms of speech, see more below. For useful discussions of Frankfurt's initial conception, see Gjelsvik (2018), Hardcastle and Reisch (2006), and Meibauer (2016); for the most systematic taxonomy of bullshit‐like insincere speech, see Heffer (2020, 2021).

^7^
Similarly, Cassam (2019, pp. 80–81) conceives of bullshit as a form of “epistemic insouciance”.

^8^
As we shall see, exploitative bullshit turns out to be far more harmful, since there is far more at stake than merely such general alethic insouciance.

^9^
Which was broadcasted, see here: https://www.youtube.com/watch?v=pSFfTG42Jl8.

^10^
For a similar example, see Kimbrough (2006, pp. 12–13).

^11^
One of the few exceptions is Heffer's (2020) discussion of bullshit as an epistemically irresponsible form of insincerity.

^12^
In particular, from “bald‐faced” or “evasive” cousins of bullshit and “misspeaking” (see note 6 above). But the two features above also delineate bullshit from “white lies” (Bok, 1989), and more importantly, from disinformation, or the dissemination of non‐randomly misleading or intentionally deceptive information (see Fallis, 2015a; Fetzer, 2004; Søe, 2021). If we hold on to the almost universally accepted claim that conventional lying requires that the speaker believes his statement to be false, *pace* Wreen (2013), who also insists on this untruth condition, bullshit cannot be reduced to conventional lying either.

^13^
This is also pointed out at various places in Heffer (2020). The same goes for discussions of other non‐alethic speech, and in particular bald‐faced lies.

^14^
It is important to note that this only applies to the cases I'm interested in, that is, those that arise in tandem with epistemic exploitation. But not all bullshit is produced strategically to this effect; much bullshit is, politically at least, rather innocuous. On politically relevant cases of bullshit, without the context of epistemic injustice, however, see again Cassam (2021).

^15^
Accordingly one‐sided, not to say naïve, are the brief reflections by Frankfurt on why bullshit is so often produced in the first place (1986, pp. 63–67). Frankfurt also fails to account for the truly social, let alone political consequences of untruthful speech (of both bullshitting and lying) in his later book *On Truth*, where he mainly considers the value of truth in practical or instrumental terms (Frankfurt, 2006, esp. chap. 6).

^16^

https://renieddolodge.co.uk/why-im-no-longer-talking-to-white-people-about-race [01.04.2021], later published as Eddo‐Lodge (2017).

^17^
Berenstain cites several other illustrative passages from essays or blogs by people of color such as Toni Morrison or Audre Lorde.

^18^
For overviews of the by now large literature, see McKinnon (2016) and Pohlhaus (2017), and for articles on more specific discussions Kidd, Medina, and Pohlhaus (2017).

^19^
I come back to testimonial injustice below.

^20^
See Dotson (2014) and Pohlhaus (2017, p. 14) helpfully distinguishes three forms and degrees of epistemic exclusion, according to how and which epistemic practices would need to be changed in order to reintegrate the contribution of the oppressed into a shared epistemic space.

^21^
For a critical discussion of the distinction between interpersonal and institutional aspects of epistemic oppression—an issue I return to in the context of EEB—see again Pohlhaus (2017).

^22^
I will not define the concept of exploitation in general; for a discussion see Zwolinski and Wertheimer (2016). Suffice to say that exploitation is essentially determined by someone being unfairly treated or privileged at the expense of some others, who are typically vulnerable. Notice though that both parties may well benefit from the exploitation, relative to their starting point, and hence we must distinguish unilaterally harmful from mutually beneficial exploitation. Furthermore, exploitation may either happen in a direct transaction (such as in unfair trade) or be structurally inscribed in institutional practices or socioeconomic systems, or both. As should become clear, epistemic exploitation can be both transactional and structural, and is typically both, but it is never mutually beneficial.

^23^
See more on the emotional dimension of this process, or on so‐called “affective injustice” in Archer and Mills (2019), Gallegos (2021), Srinivasan (2018), and Whitney (2018). Below, I argue that Sartrean EEB additionally points to what I call “emotional hostage‐taking” which is germane to but different from what is discussed in this recent work.

^24^
Further ones include “gaslighting” (Abramson, 2014; see Berenstain, 2016, p. 580) and “extracted speech” (McKinney, 2016), which are, however, less relevant for Sartrean EEB.

^25^
For a related form of self‐censoring, see Lee's (2021) notion of “anticipatory epistemic injustice”.

^26^
This is certainly not to say that anti‐Semitic discourse would be more dangerous than other racist or anti‐minoritarian discourses. Such comparisons are not only futile but dangerous. The point is rather that any discourse that has the characteristics of epistemically exploitative bullshit (which I present here merely by way of example with reference to the Sartrean anti‐Semite) is precisely more dangerous and destructive than bullshit or epistemic exploitation *in isolation*.

^27^
Given the more directly exploitative and hostile features of this discourse, it's only a rather harmless result of this insouciance that it “favors laziness of mind” (Sartre, 1995b[1946]], p. 43), however much such laziness is a serious enough epistemic vice, eventually leading to more harmful epistemic behavior, as contemporary critical epistemologists writing on bullshit and epistemic injustice have rightly pointed out (Cassam, 2019; Medina, 2013).

^28^
The English translation is imprecise here, translating *désorienter* (Sartre, 1995b[1946], p. 24) as “disconcert” and thereby missing out on the intentional and epistemologically relevant aspect of insincerity I wish to stress here.

^29^
And, as Sartre adds, also to “intimidate” them; I will return to this point later.

^30^
Sartre explicitly claims that the anti‐Semite is (acting) in bad faith in several passages of his essay; beyond the motto, see, for example, 1995b[1946], p. 27, 32f. and 40. Incidentally, I will not discuss Sartre's controversial claims regarding the (in‐)authenticity of the Jew in reaction to anti‐Semitism (see e.g., ibid., p. 99); for critical analyzes, see Consoni and Liska (2020), McBride (1991), Walzer (1995), and Yinon (2020); for some contextualizing discussions of the authenticity in Sartre's ethical and political thought, see Daigle (2011) and Heter (2006).

^31^
Obviously, I cannot discuss the exceedingly complex nature of Sartrean bad faith and the puzzles it poses in due detail. I restrict my discussion only to those aspects which support my claim that bad faith is pertinent to EEB; for a more complex, and complete, picture, see Webber (2009), chaps. 6 and 7.

^32^
Which was written just a year after the publication of *Being and Nothingness*, in which Sartre most systematically explored bad faith, after having introduced the idea in his early essay *The Transcendence of the Ego* (Sartre, 1991[1936/37]).

^33^
And it is also this basic fear of truth (as such and about oneself) that leads to the antagonistic and genuinely affective relationship to the other, or to hatred: “The anti‐Semite has chosen hate because hate is a faith” (Sartre, 1995b[1946]], p. 19)—namely, a “bad” or “false” one. I cannot discuss the affective aspects of EEB here; for a detailed analysis of hatred, with cursory reference to Sartre, see Szanto (2020).

^34^
I have characterized this process as the “negative dialectics” and “negative solidarity” of ingroup‐alignment and outgroup exclusion and stereotyping and discussed it (partly in relation to Sartre) in Szanto (2020) and Szanto (2022). See more on this process below. On how bad faith is involved in racial reification and essentialization, see also van Leeuwen (2008) (which represents a sustained criticism of Gordon's (1995) earlier account of bad faith and racism); see also Martin (2002).

^35^
This is yet another crucial *social* aspect in Sartre's theory of bad faith, which has so far not been sufficiently recognized; for different emphases on the sociality of bad faith (as conceived in *Being and Nothingness*), see Eshleman (2008), and cf. Santoni (2008), and again Webber (2009). More generally on *collaborative* forms of self‐deception and akrasia, see my proposal in Szanto (2017).

^36^
It would be interesting to discuss these mechanisms of bad faith in relation to similar mechanisms of self‐categorization, self‐stereotyping underlying group‐identification and prejudice as described by social psychologists, and in particular social identity theories (Allport, 1954; Hogg & Abrams, 1988; Tajfel, 1981).

^37^
For discussion, see the references in Fn. 28 above.

^38^
For Sartre's related discussion of the “racism of the Other” and “serial responsibility” for racist violence, see Sartre (2006[1960], pp. 652–654).

^39^
See also ibid., 20–22.

^40^
For an analysis of the failures of performative speech acts *of the victims* of epistemic injustice, see Kukla (2014).

^41^
Nor, for that matter, do I wish to level the difference between explicit prejudices and implicit biases, which, if we want to analyze the mechanisms of epistemic oppression, would need to be considered more thoroughly than I could in this paper.

^42^
Williams also recognized that there is an essential relation between self‐deception, other‐deception and failures of epistemic accuracy when he notes that “our failure as self‐deceived are to be found at least as much in our lack of epistemic prudence as victims as in our insincerity as perpetrators […] when we consider […] the *passion for getting it right*—then we must remember equally the role of Sincerity in one's dealing with oneself.” (Williams, 2002, p. 126).
